# Curvature-Based Environment Description for Robot Navigation Using Laser Range Sensors

**DOI:** 10.3390/s90805894

**Published:** 2009-07-24

**Authors:** Ricardo Vázquez-Martín, Pedro Núñez, Antonio Bandera, Francisco Sandoval

**Affiliations:** 1 Departamento de Tecnología Electrónica, University of Málaga, E.T.S.I. Telecomunicación, Campus Teatinos, Málaga, Spain; E-Mails: rvmartin@uma.es; ajbandera@uma.es; fsandoval@uma.es; 2 Departamento de los Computadores y las Comunicaciones, University of Extremadura, Escuela Politécnica, Cáceres, Spain

**Keywords:** laser scan data segmentation, mobile robot navigation, adaptive curvature estimation

## Abstract

This work proposes a new feature detection and description approach for mobile robot navigation using 2D laser range sensors. The whole process consists of two main modules: a sensor data segmentation module and a feature detection and characterization module. The segmentation module is divided in two consecutive stages: First, the segmentation stage divides the laser scan into clusters of consecutive range readings using a distance-based criterion. Then, the second stage estimates the curvature function associated to each cluster and uses it to split it into a set of straight-line and curve segments. The curvature is calculated using a triangle-area representation where, contrary to previous approaches, the triangle side lengths at each range reading are adapted to the local variations of the laser scan, removing noise without missing relevant points. This representation remains unchanged in translation or rotation, and it is also robust against noise. Thus, it is able to provide the same segmentation results although the scene will be perceived from different viewpoints. Therefore, segmentation results are used to characterize the environment using line and curve segments, real and virtual corners and edges. Real scan data collected from different environments by using different platforms are used in the experiments in order to evaluate the proposed environment description algorithm.

## 1. Introduction

Extracting useful information from the environment has an important effect on the robot navigation process. Simultaneous localization and map building (SLAM), path planning, or even a virtual reconstruction of the scene for supervising the robot navigation are different examples where a detailed description of the environment can usually improve their results. To address this issue, an appropriate representation of the working environment of the mobile robot must be acquired, which is not trivial. Many factors and physical constraints affect the reliability of such representation [1].

One of the first tasks in the navigation system design is to determine the type of sensor required to obtain the desired description in a particular environment. The most appropriate sensor for the application depends on the size of the operation area, the environmental conditions, and the required representation level. Indeed, the most important factor that determines the quality of the representation is this external sensor, and above all, its accuracy. With regard to the mobile robotic tasks, an accurate localization in known or unknown environments is essential for autonomous mobile robot navigation. Pure dead-reckoning methods such as odometry are prone to drift, and an estimate is needed to reduce the growing unbounded errors [2]. In order to provide a precise position estimation, external sensors, like sonar or laser range finder sensors, are extensively used in robotics, especially in indoor environments [3–6]. In these sensors, the accuracy is a function of their specifications and the type of features used to represent the environment. Other kinds of commonly used sensors in robotics are cameras, more specifically, monocular, stereo, or trinocular vision systems [7–12]. In these cases, the accuracy of the sensor is a function of the captured image resolution and the features used in the representation.

**Figure 1.**
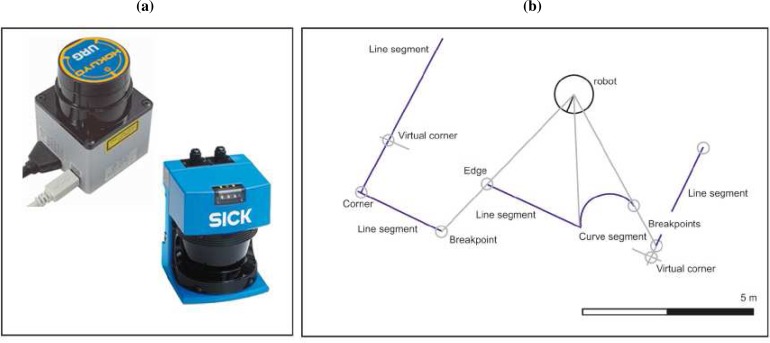
(a) Two laser range sensors widely used in Robotic: a LMS200 from SICK and a HOKUYO URG-04LX. (b) Natural landmarks detected and characterized in this work: breakpoints, rupture points, line and curve segments, corners and edges.

In general, the structural features commonly found in the environment are assumed to be invariant to height (e.g., walls, corners, columns). Using this assumption, a planar representation would be adequate for feature extraction and a distance-based sensor can be used. Among different types of sensors, 2D laser range finders have been increasing popular during the last decade, because they provide dense and accurate range measurements with high angular resolution and sampling rates. Figure 1(a) illustrates two classical laser range sensors used in robotics: a LMS200 from SICK, and a HOKUYO URG-04LX. On the other hand, in terms of cost, it is an affordable device for most mobile robotics systems.

Once the sensor is chosen, the second task that we must address is to match the obtained data with the expected data available in a map. To this end, two approaches have been used in mobile robotics: point-based and feature-based matching. Feature-based approaches increase the efficiency and robustness of this process by transforming the acquired raw sensor data into a set of geometric features. Because they are more compact, these feature-based approaches require much less memory than the point-based approaches and can still provide rich and accurate information [13]. Besides, these methods are more robust to the noise resulted from spurious measurements and unknown objects. Thus, feature-based model is a typical choice for the map representation, which allows the use of multiple models to describe the measurement process for different parts of the environment.

This work extends the CUrvature-BAsed (CUBA) approach for environment description: a feature-based approach proposed by Núñez *et al*. [14–16]. In these previous works, the authors present a feature-based approach which employs multiple models to characterize the environment. Specifically, the laser scan is analyzed to detect rupture points, breakpoints and four types of landmarks: line segments, corners, center of curve segments and edges [14–16] [see Figure 1(b)]. With respect to these previous works, a new laser scan data segmentation based on curvature information is proposed. In order to improve the robustness against noise, this curvature is calculated using a triangle-area representation where the triangle side lengths at each range reading are adapted to the local variations of the laser scan, removing noise without missing relevant points. Besides, in this paper, the proposed environment representation has been used inside a SLAM approach based on the Extended Kalman Filter (EKF).

This work has been organized as follows: Firstly, the most popular methods available in the literature for laser scan data segmentation are briefly described in Section 2. Next, a multi-scale method based on the curvature estimation of the scan data is presented in Section 3. Section 4 describes some improvements to the proposed segmentation module of the approach which have been included in order to increase its robustness against noise and its invariance to translation and rotation. Experimental results and a brief discussion have been included in Sections 5 and 6, respectively. Finally, a brief glossary is given, which includes a list of words related to the robotics field.

## 2. Laser Scan Data Segmentation Algorithms

### 2.1. Problem Statement

**Figure 2.**
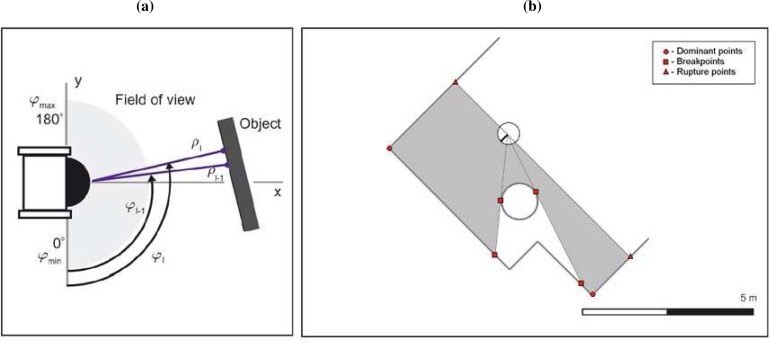
(a) Scan reference frame variables. (b) Problem statement.

Scan data provided by 2D laser range finders are typically in the form {(*r*, *φ*)_*i*|*i*=1...*N*_*R*__}, on which (*r*, *φ*)*_i_* are the polar coordinates of the *i*th range reading (*r_i_* is the measured distance of an obstacle to the sensor rotating axis at direction *φ_i_*), and *N_R_* is the number of range readings. Figure 2(a) represents all these variables. It can be assumed that the noise on both measurements, range and bearing, follows a Gaussian distribution with zero mean and variances $\sigma_{r}^{2}$ and $\sigma_{\varphi }^{2}$, respectively. The aim of segmenting a laser scan is to divide it into clusters of range readings associated to different surfaces, planar or curves, of the environment. There are two main problems in laser scan segmentation:

- How many segments are there?
- Which range readings belong to which segment?

In order to establish the limits of these segments, these problems can be stated as the search for the range readings associated to the discontinuities in the scanning process or to the changes in the orientation of the scan [see Figure 2(b)].

To detect these changes, two main types of techniques have been proposed in the literature. The most popular ones try to find specific geometric features in the scan. Specifically, polygonal approximation techniques originated from computer vision have been widely used to deal with office-like environments, which can be described using line segments. This segmentation process is achieved by checking some heuristic line criteria (i.e., error bound) while concatenating consecutive points. On the other hand, the laser scan data can be represented by a local descriptor which can be analyzed to extract the set of dominant points which correctly segments the scan into curve and line segments.

### 2.2. Polygonal Based Methods

Among the polygonal-based techniques, the incremental and split-and-merge (SM) approaches are probably the most popular and simple line segments extractors. The split-and-merge algorithm fits a line segment to the set of range readings, and it then divides this line into two new line segments if there is a range reading whose distance to the line is greater than a given threshold. This splitting process is then iteratively applied to the newly generated line segments. Finally, when all line segments have been checked, collinear segments are merged. This algorithm has been used to extract line segments in many robotic research [17–20]. The incremental algorithm, also known as *Line-Tracking*, starts with two close points and adds the next scan point to the end of the segment when a predefined line condition is satisfied. If the criterion is not achieved, the current line is finished and a new line is started in the next point.

Similar to the SM algorithm, the iterative-end-point-fit method (IEPF) provides a polygonal approximation to the laser scan at a low cost [18]. The procedure is similar to the first part of the SM algorithm. A line is fitted to a set of scan points simply by connecting the end points of two sets. The point with the maximum distance to the line is detected and the set is split up if the distance exceeds a fixed threshold. This splitting process is repeated until the maximum distance is lower than the threshold for all sets.

In order to avoid the need to guess the number of initial clusters, Borges and Aldon [19] employ fuzzy clustering in a split-and-merge framework. The split phase is based on the IEPF method, where for each iteration a set of scan points is divided into two sets if a threshold established for a dispersion measure is not satisfied. Unlike IEPF, the obtained set does not obey the acquired order of the points. In the merge phase, the two closest lines to a reference line are selected as fusion candidates. The fused line is the one that gives the smallest dispersion with the reference line and the given threshold for a single line is fulfilled.

Other model-based popular approaches are based on the Hough transform. The Hough transform has been successfully applied to detect lines in intensity images and it has been brought into robotics for achieving this same aim for scan images [21, 22]. The set of scan data points is sorted into subsets of roughly collinear points using the Hough transform that is based on a voting strategy to determine the best fit for the data subset. The main drawback of this method is the difficulty in choosing a correct size for the voting grid. The parametric space must be discretized and the accuracy is highly affected in real time applications. In order to avoid this problem, the approach proposed by Bandera *et al*. [23] employs a variable bandwidth mean shift algorithm to independently cluster the items of the parameter space in a set of classes.

Finally, the aim of *Random Sampling Segmentation Algorithms* is to find a suboptimal probabilistic model to classify the data points and to separate inliers from outliers. Usually, RANdom SAmpling Consensus (RANSAC) is used to detect outliers in a set of data, because it is an efficient algorithm for robust fitting of any kind of models in the presence of data outliers. In this scheme, an algorithm for robust data segmentation is presented in [24]. It is adapted to scale space by using the Adaptive Scale Sample Consensus (ASSC), a modification of RANSAC involving an adaptive scale estimation. The ASSC is a kernel-based scale estimator based on the mean shift method for the data driven scale estimate.

### 2.3. Curvature-Based Methods

**Figure 3.**
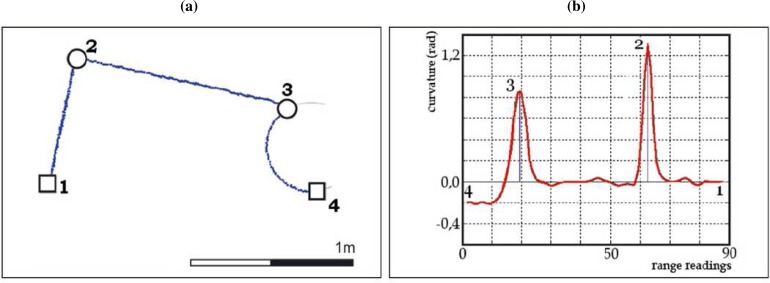
(a) Segment of a single laser scan (□-breakpoints, o-corners). (b) Curvature function associated to (a).

Curvature functions basically describe how much a curve bends at each point. Peaks of the curvature function correspond to the corners of the represented curve and their height depends on the angle at these corners. Flat segments whose average value is larger than zero are related to curve segments and those whose average value is equal to zero are related to straight line segments. Figure 3(a) presents a curve yielding two corners (points 2 and 3) and a curve segment (from point 3 to 4). Peaks corresponding to 2 and 3 can be appreciated in its curvature function [Figure 3(b)]. It also shows that segment 3–4 has an average value greater than zero, but it is not flat due to noise. Nevertheless, peaks in that segment are too low to be considered as the corners of the curve. Finally, segments 1–2 and 2–3 present a curvature average value near to zero, as expected in line segments.

In a general case, the curvature *κ*(*t*) of a parametric plane curve, *c*(*t*) = (*x*(*t*), *y*(*t*)), can be calculated as [25, 26]

(1) $$\kappa (t)=\frac{\dot{x}(t)\ddot{y}(t)-\ddot{x}(t)\dot{y}(t)}{{\left(\dot{x}{\left(t\right)}^{2}+\dot{y}{\left(t\right)}^{2}\right)}^{3/2}}$$

This equation implies that estimating the curvature involves the first and second order directional derivatives of the plane curve coordinates, (*ẋ*, *ẏ*) and (*ẍ*, *ÿ*), respectively. This is a problem in the case of computational analysis where the plane curve is represented in a digital form [26]. In order to solve this problem, two different approaches have been proposed:

- Interpolation-based curvature estimators. These methods interpolate the plane curve coordinates and then differentiate the interpolation curves. Thus, Mokhtarian *et al*. [25] propose to filter the curve with a one-dimensional Gaussian filter. This filtering removes the plane curve noise.
- Angle-based curvature estimators. These methods propose an alternative curvature measure based on angles between vectors, which are defined as a function of the discrete curve items. Thus, the curve filtering and curvature estimation are mixed by Agam *et al*. [27], which define the curvature at a given point as the difference between the slopes of the curve segments on the right and left side of the point, where slopes are taken from a look-up table. The size of both curve segments is fixed. Liu *et al*. [28] compute the curvature function by estimating the edge gradient at each plane curve point, which is equal to the arctangent of its Sobel difference in a 3×3 neighborhood. Arrebola *et al*. [29] define the curvature at a given point as the correlation of the forward and backward histograms in the *k*-vicinity of the point, where the resulting value is modified to include concavity and convexity information.

Due to the characteristic noise associated to the curvature estimation, all these algorithms implicitly or explicitly filter the curve descriptor at a fixed cut frequency to remove noise and provide a more robust estimation of the curvature at each plane curve point (*single scale methods*). However, features appear at different natural scales and, since most methods filter the curve descriptor at a fixed cut frequency, only features unaffected by such a filtering process may be detected. Thus, in the case of angle-based curvature estimators, algorithms described above basically consist of comparing segments of *k*-points at both sides of a given point to estimate its curvature. Therefore, the value of *k* determines the cut frequency of the curve filtering. In these methods, it is not easy to choose a correct *k* value: when *k* is small, the obtained curvature is very noisy and, when *k* is large, corners which are closer than *k* points become missing. To avoid this problem, some methods propose iterative feature detection for different cut frequencies, but they are slow and, in any case, they must choose the cut frequencies for each iteration [30]. Another solution is to adapt the cut frequency of the filter at each curve point as a function of the local properties of the shape around it [31].

Both approaches have been applied to laser scan segmentation. Thus, the iterative curvature scale space (CSS) was used by Madhavan and Durrant-Whyte [32] to extract stable corners. This algorithm convolves the curve descriptor with a Gaussian kernel and imparts smoothing at different levels of scale (the scale being proportional to the width of the kernel). From the resulting curve descriptor, features associated to the original shape can be identified [25]. In order to achieve a robust determination of dominant points, the algorithm detects them at the most coarse scale *σ_max_*, but localizes the dominant point position at the finest scale *σ_min_*. In order to avoid a slow iterative estimation of the curvature, an adaptive algorithm was employed by Núñez *et al*. [16] to extract corners, line and curve segments from the laser scan data.

## 3. CUrvature-BAsed Environment Description Framework

The environment description algorithm described in this paper is divided in two main stages. The first one is a segmentation stage, which divides the scan data acquired by the laser range sensor to a set of point clusters associated to line or corner segments. The next stage detects and characterizes natural landmarks (i.e. line and curve segments, corners and edges) according to these clusters, through both extracting their pose and estimating the uncertainties. This complete characterization allows the use of these features on a later robotic navigation tasks (e.g., SLAM). This work is based on previous papers [14, 16]. In order to improve the robustness against noise in the segmentation stage, this paper includes a novel technique for affine invariant curvature estimation. The new segmentation module is described in the Section 4.

### 3.1. Segmentation Algorithm

In this section, the segmentation algorithm developed inside the CUrvature BAsed environment description framework (CUBA in short) is presented. Instead of using a slow, iterative approach, dominant points can be robustly detected by adapting the scale to the local surroundings of each range reading. This solution has been adopted by Núñez *et al*. [14, 16]. The adaptive curvature approach allows to rapidly segment the laser scan into curve and line segments.

**Figure 4.**
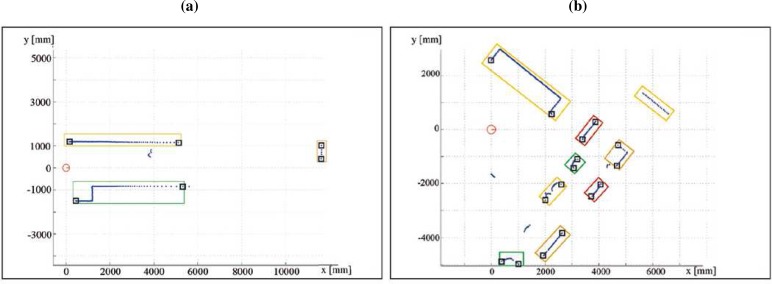
(a)–(b) Laser scan and extracted breakpoints (squares). It must be noted that segments of the laser scan which present less than ten range readings are not taken into account (they are marked without boxes in the figures).

**Figure 5.**
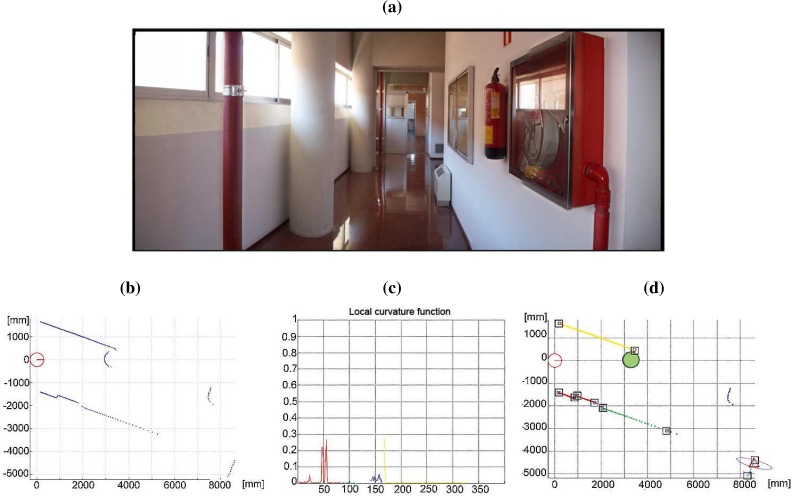
(a) A real environment where CUBA algorithm has been tested; (b) scan data acquired by the laser range sensor; (c) curvature function associated to (a) using the method proposed in [16]; and (d) natural landmarks (*triangle*-corners, *square*-end-points of line segments, o-circles) with their associated uncertainties (ellipses in the images).

In this approach, the segmentation is achieved in two consecutive steps. The first type of segmenting points may arise from the absence of obstacles in the scanning direction (*rupture points*) or from the change of surface being scanned by the sensor (*breakpoints*) [19]. Rupture points cannot be detected by making inferences about its possible presence. They indicate a discontinuity during the measurement and its presence must be informed by the range finder [14]. On the contrary, breakpoints are detected by making inferences about the possible presence of discontinuities in a sequence of valid range data [14, 19]. Basically, the aim of a breakpoint detector is to verify if there exists a discontinuity between two consecutive range readings (*r*, *φ*)*_n_* and (*r*, *φ*)_*n*−1_. This algorithm allows to reject isolated range readings, but it leads to an under-segmentation of the laser scan, i.e., extracted segments between breakpoints typically group two or more different structures (see Figure 4). In order to avoid this problem, once the whole laser scan is divided into sets of consecutive range readings, a second segmentation criterion is applied into each set. This approach is focused on the correct selection of the set of dominant points present into a part of the scan bounded by two consecutive breakpoints. If the whole laser scan is divided into different sets of consecutive range readings by the breakpoint detector, this specific problem can be stated as the estimation of the curvature function associated to each set. Therefore, this one is based on the curvature associated to each range reading: consecutive range readings belong to the same segment while their curvature values are similar. To perform this segmentation task, the adaptive curvature function associated to each segment of the laser scan is computed [16]. Then, this information is employed to segment the laser scan into clusters of homogeneous curvature. The whole process to achieve this segmentation task is shown with details in [16]. Figure 5 (a) shows a real environment used to illustrate the CUBA framework. The scan data provided by the sensor and the curvature estimates by the segmentation stage are drawn in Figures 5 (b) and 5 (c), respectively.

### 3.2. Natural Feature Extraction and Characterization

As can be seen in Figure 5 (c), the segmentation algorithm can directly provide two different natural features: line and curve segments [16]. In order to include these items as features in a compact form to be used in a subsequent process, it is necessary to characterize them by a set of invariant parameters, and moreover, to estimate their uncertainties. This is typically achieved by fitting parametric curves to measurement data associated to each line or curve segment and by evaluating the uncertainty associated to the measured data. Thus, line and curve segments can be used as stable features. Finally, other types of features can be extracted and characterized as corners or edges. The method used to characterize these natural landmarks is based on our previous work [14]. This section introduces the method for extracting and characterizing natural features from the segmented laser data.

**Figure 6.**
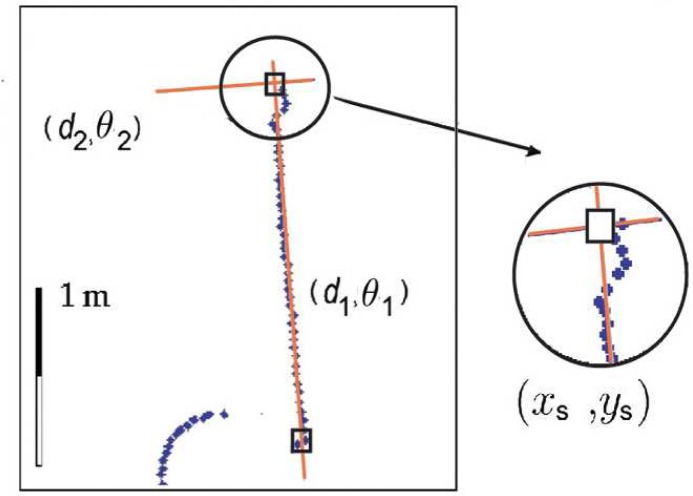
A real corner is not usually located at one of the laser range readings (they are marked as blue dots over the detected line segments).

**Figure 7.**
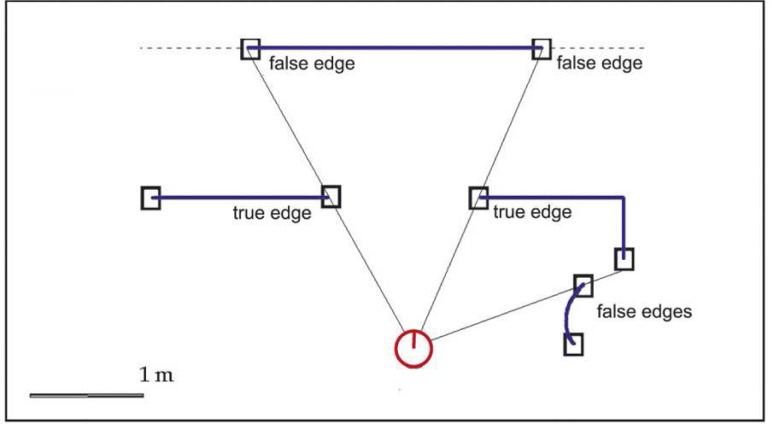
An edge is defined as a breakpoint associated to the end-point of a plane surface which is not occluded by any other obstacle.

1. Line segments
  In order to provide precise feature estimation it is essential to represent uncertainties and to propagate them from single range reading measurements to all stages involved in the feature estimation process. As previously mentioned, the methods try to fit parametric curves to each segmented data. An approach for line fitting is to minimize the sum of square perpendicular distances of range readings to lines. This yields a nonlinear regression problem which can be solved for polar coordinates [33]. The line in the laser range finder’s polar coordinate system is represented as
  (2) $$r=\frac{d}{\text{cos}(\theta -\phi )}$$
  where *θ* and *d* are the line parameters in the normal form representation:
  (3) x cos θ+y sin θ=d
  being *θ* the angle between the *X* axis and the normal of the line and *d* the perpendicular distance of the line to the origin. Then, the orthogonal distance *d_i_* of a range reading, (*r*, *ϕ*)*_i_*, to this line is
  (4) $$d_{i}=r_{i}\;\text{cos}(\theta -\phi_{i})-d$$
  Under the assumption of known uncertainties, a weight for each measurement point can determine and fit the line in the generalized least squares sense, whose solution is (see [14] for further details)
  (5) $$\begin{array}{r} \theta =\frac{1}{2}\text{arctan}\;\left(\frac{\sum_{i}r_{i}^{2}\;\text{sin}\;2\phi_{i}-\frac{2}{n}\sum_{i}\;\sum_{j}r_{i}r_{j}\;\text{cos}\;\phi_{i}\;\text{sin}\;\phi_{j}}{\sum_{i}r_{i}^{2}\;\text{cos}\;2\phi_{i}-\frac{1}{n}\sum_{i}\sum_{j}r_{i}r_{j}\;\text{cos}(\phi_{i}+\phi_{j})}\right)\\ d=\frac{\sum_{i}r_{i}\;\text{cos}\;(\phi_{i}-\theta )}{n} \end{array}$$
  Figure 5 (d) presents the detected landmarks corresponding to the scan data acquired by the sensor in 5 (b). In this case, Figure 5 (d) shows the line segments extracted using the described approach (end-points of the line segments are illustrated as squares). These end-points are determined by the intersection between this line and the two lines which are perpendicular to it and pass through the first and last range readings.
2. Curve segments
  Although many circle fitting methods have been proposed, it is a common choice to achieve this by minimizing the mean square distance from the data points to the fitting circle. Basically, the Least Squares Fit (LSF) assumes that each data point is the noised version of the closest model point. This assumption is valid when data points are not contaminated with strong noise.
  Let the data points be {*x_i_*, *y_i_*}_|*i*=1...*m*_ (*m* > 3), with an uncertainty ellipse specified in terms of the standard deviations *p_i_* and *q_i_*, and the correlations *r_i_*. The problem is to obtain the center (*x_c_*, *y_c_*) and the radius *ρ* of the circle *C* which yields the best fit to this data. It is also required to determine the variance matrix associated to the circle parameters.
  This problem is stated as the minimization of the difference between the set of points {*x_i_*, *y_i_*} and their corresponding points {*x_c_* + *ρ* cos *ϕ_i_*, *y_c_* + *ρ* sin *ϕ_i_*} which lie on *C*. This difference is summarized by the 2*m*-element error vector *ε*:
  (6) $$\begin{array}{c} \varepsilon ={(x_{1}-(x_{c}+\rho \;\text{cos}\;\phi_{1}),y_{1}-(y_{c}+\rho \;\text{sin}\;\phi_{1}),\ldots ,x_{m}-(x_{c}+\rho \;\text{cos}\;\phi_{m}),y_{m}-(y_{c}+\rho \;\text{sin}\;\phi_{m}))}^{T}\\ ={\left(\Delta x_{1},\Delta y_{1},\ldots \Delta x_{m},\Delta y_{m}\right)}^{T} \end{array}$$
  This error vector has the known 2*m*×2*m* block diagonal variance matrix *V* = *diag*(*V*_1_...*V_m_*), where
  (7) $$V_{i}=\left[\begin{array}{cc} p_{i}^{2} & r_{i}\\ r_{i} & q_{i}^{2} \end{array}\right]$$
  Then, assuming that the errors are normally distributed, the *maximum likelihood* (ML) problem consists of minimizing
  (8) $$\mathrm{minimize}\;\varepsilon^{T}\;V^{-1}\varepsilon$$
  with respect to the vector *b* = (*ϕ*_1_, ..., *ϕ_m_*, *x_c_*, *y_c_*, *ρ*)*^T^*.
  In order to solve the minimization problem, the classical Gauss-Newton algorithm with the Levenberg-Marquardt correction [34, 35] is used. This algorithm finds the vector *b* which minimizes 8 in an iterative way. It approximates the objective function with the square of the norm of a linear function. Thus, at each iteration, the linear least-squares problem is solved
  (9) $$\min_{\delta b}{||{\varepsilon^{\prime }}^{\left(k\right)}-\nabla {\varepsilon^{\prime }}^{\left(k\right)}\cdot \delta b||}_{2}$$
  where ∇*ε*′^(*k*)^ is the Jacobian matrix of first partial derivatives of *ε*′ with respect to *b* and *ε*′^(*k*)^ is *ε*′, both evaluated at *b*^(*k*)^. A detailed description of the Levenberg-Marquardt algorithm can be found at [35]. In this case, the starting estimate for the centre coordinates and radius is obtained using the Taubin’s approximation to the gradient-weighted algebraic circle fitting approach [34].
3. Finally, to obtain the variance matrix of the center coordinates and radius, an estimate of the variance matrix of the vector *b* must be obtained. Further details about the fitting problem are shown in [15]. Figure 5 (d) draws the circle segment extracted using the described approach for the scan data provided by the sensor in Figure 5 (b).
4. Real and virtual corners
  As pointed out by Madhavan and Durrant-White [32], one of the main problems of a localization algorithm only based on corner detection is that the set of detected natural landmarks at each time step can be very limited, specially when it works on semi-structured environments. This generates a small observation vector that does not provide enough information to estimate the robot pose. To address this problem, the description algorithm described in this paper uses real and virtual corners as natural landmarks of the robot environment. Real corners are due to change of surface being scanned or change in the orientation of the scanned surface. Thus, they are not associated to laser scan discontinuities. On the other hand, virtual corners are defined as the intersection of extended line segments which are not previously defined as real corners. In order to obtain the corner location, it must be taken into account that failing to identify the correct corner point in the data can lead to large errors that increase with the distance to the detected corner (see Figure 6). Therefore, it is usually not a good option to locate the corner in one of the scan range readings. Another choice is to extract the corner location as the intersection of the two associated lines. Thus, the corner can be detected as the farthest point from a line defined by the two non-touching endpoints of the lines or by finding that point in the neighborhood of the initial corner point, which gives the minimum sum of error variances of both lines [36]. The existence of a corner can be determined from the curvature function [16], but its characterization (estimation of the mean pose and uncertainty measurement) is conducted using the two lines that generates the corner [14]. Figure 5 (d) illustrates the virtual corner detected by the algorithm (triangle) for the real scene described in Figure 5 (a). The associated covariance matrix has been also represented (ellipse).
5. Edges
  The adaptive breakpoint detector searches for large discontinuity values in the laser scan data. Range readings that define this discontinuity are marked as breakpoints. Edges are defined as breakpoints associated to end-points of plane surfaces [37]. To satisfy this condition, the portion of the environment where the breakpoint is located must be a line segment and must not be occluded by any other obstacle. This last condition is true if the breakpoint is closer to the robot than the other breakpoint defined by the same large discontinuity (see Figure 7). It must be also noted that, when the laser range finder does not work with a scanning angle of 360°, the first and last breakpoints will not be considered as edges, because it is impossible to know if they define the end-point of a surface.
  Edges are characterized by the Cartesian position (*x*, *y*) of the breakpoint and by the orientation of the plane surface described by the line segment, *θ* ([14]).

## 4. Affine-invariant Laser Scan Segmentation

The CUBA algorithm is a curvature based approach to extract dominant points from the laser scan data. After the segmentation process a set of corners, line and curve segments are obtained and characterized to be used as natural landmarks. Although the adaptive curvature estimation provides a robust criterion to the laser scan segmentation, some aspects can be considered to improve this algorithm to be more robust against noise and affine transformations. This section describes the new technique proposed in this paper for segmenting the scan data acquired by a laser range sensor.

### 4.1. Adaptive Estimation of the Region-of-support

From the pioneering paper of Teh and Chin [38], many researchers have argued that the estimation of the curvature relies primarily on the precise calculation of the region-of-support associated to each curve point. In the described framework, in order to specify the region-of-support associated to the range reading *i* of the laser scan, the algorithm must determine the maximum length of scan that presents no significant discontinuities on the right and left sides of the range reading *i*, *t_f_* [*i*] and *t_b_*[*i*], respectively. To estimate the *t_f_* [*i*] value, the algorithm first computes two sets of triangles, ${\left\{t_{j}^{a}\right\}}_{j=i}^{i+t_{f}\;[i]-1}$ and ${\left\{t_{j}^{c}\right\}}_{j=i}^{i+t_{f}\;[i]-1}$. The area of the triangle $t_{j}^{a}$ is defined as

(10) $$|t_{j}^{a}|\;=\;\frac{1}{2}\left|\begin{array}{ccc} x_{j} & x_{c} & x_{j+1}\\ y_{j} & y_{c} & y_{j+1}\\ 1 & 1 & 1 \end{array}\right|$$

where (*x_j_*, *y_j_*) and (*x*_*j*+1_, *y*_*j*+1_) are the Cartesian coordinates of the arc range readings *j* and *j* + 1 and (*x_c_*, *y_c_*) is the robot position, **x***_c_*.

The area of the triangle $t_{j}^{c}$ is defined as

(11) $$|t_{j}^{c}|=\frac{1}{2}\left|\begin{array}{ccc} x_{j}^{p} & x_{c} & x_{j+1}^{p}\\ y_{j}^{p} & y_{c} & y_{j+1}^{p}\\ 1 & 1 & 1 \end{array}\right|$$

where ( $x_{j}^{p}$, $y_{j}^{p}$) is the projection of (*x_j_*, *y_j_*) on the chord that joins the range readings *i* and *i* + *t_f_* [*i*].

If $T_{i,t_{f}\;[i]}^{a}$ and $T_{i,t_{f}\;[i]}^{c}$ are equal to $\sum_{j=i}^{i+t_{f}\;[i]-1}\left|t_{j}^{a}\right|$ and $\sum_{j=i}^{i+t_{f}\;[i]-1}\left|t_{j}^{c}\right|$, respectively, then *t_f_* [*i*] will be defined by the largest value that satisfies

(12) $$(T_{i,t_{f}\;[i]}^{a}-(T_{i,t_{f}\;[i]}^{a}\cap T_{i,t_{f}\;[i]}^{c}))<U_{t}$$

**Figure 8.**
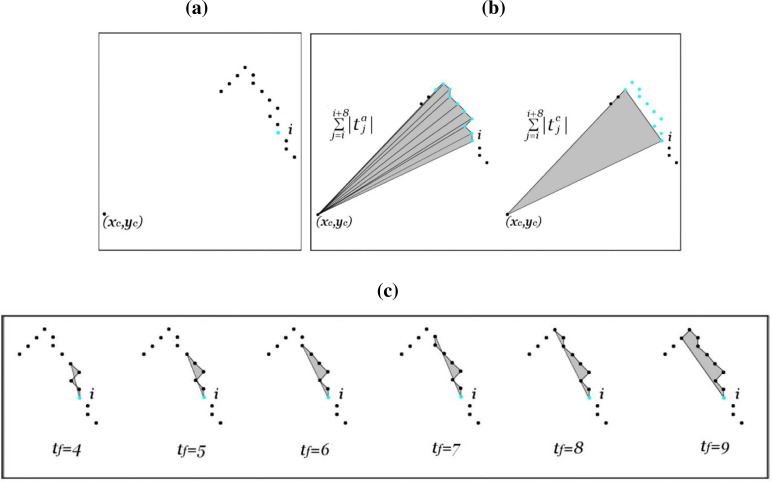
Calculation of the maximum length of contour presenting no significant discontinuity on the right side of range reading *i* (*t_f_* [*i*]): (a) Part of the laser scan and point *i*; (b) scan data acquired by the laser range sensor; and (c) evolution of the area delimited by the arc and the chord ( $\sum_{j=i}^{i+t_{f}\;[i]-1}|t_{j}^{a}|-(\sum_{j=i}^{i+t_{f}\;[i]-1}\left|t_{j}^{a}|\cap \sum_{j=i}^{i+t_{f}\;[i]-1}\left|t_{j}^{c}\right|\right)$). It can be noted that this area suffers a sharp increasing when *t_f_* [*i*] ≥ 8. This change allows to estimate the correct *t_f_* [*i*] value and it will be detected in our approach using the Equation 12 (in this case, *t_f_* [*i*] = 8).

Figure 8 shows the process to extract one *t_f_* [*i*] value. *t_b_*[*i*] is also set according to the described scheme, but using *i* − *t_b_*[*i*] instead of *i* + *t_f_* [*i*].

The correct selection of the *U_t_* value is very important. Thus, if the value of *U_t_* is large, *t_f_* [*i*] and *t_b_*[*i*] tend to be large and the contour details may be missed; if it is small, *t_f_* [*i*] and *t_b_*[*i*] are always very small and the resulting function is noisy. In order to set it correctly, a set of real plane surfaces have been scanned at different distances from the sensor. In these surfaces, this value must be fixed to not to detect any local peak. This simple experiment has provided us an *U_t_* value equal to 25.0 cm^2^, which has been successfully employed in all experiments.

### 4.2. Affine-invariant Laser Scan Segment Descriptor

Many researchers have used the area of the triangle, formed by the curve points, as the basis for shape representations [39]. The proposed laser scan segmentation algorithm employs a curvature estimator to characterize the shape contour, which is based on this triangle-area representation (TAR). Given a laser scan segment and, once this proposal has determined the local region-of-support associated to every range reading, the process to extract the associated TAR consists of the following steps:

1. Calculation of the local vectors *f⃗_i_* and *b⃗_i_* associated to each range reading *i*. These vectors present the variation in the *X* and *Y* axis between range readings *i* and *i* + *t_f_* [*i*], and between *i* and *i* − *t_b_*[*i*]. If (*x_i_*, *y_i_*) are the Cartesian coordinates of the range reading *i*, the local vectors associated to *i* are defined as
  (13) $$\begin{array}{l} {\vec{f}}_{i}=(x_{i+t_{f}\;[i]}-x_{i},y_{i+t_{f}\;[i]}-y_{i})=(f_{x_{i}},\;f_{y_{i}})\\ {\vec{b}}_{i}=(x_{i-t_{b}\;[i]}-x_{i},y_{i-t_{b}[i]}-y_{i})=(b_{x_{i}},\;b_{y_{i}}) \end{array}$$
2. Calculation of the TAR associated to each range reading. The signed area of the triangle at contour point *i* is given by [39]:
  (14) $$\kappa_{i}=\frac{1}{2}\left|\begin{array}{ccc} b_{x_{i}} & b_{y_{i}} & 1\\ 0 & 0 & 1\\ f_{x_{i}} & f_{y_{i}} & 1 \end{array}\right|$$
3. TAR Normalization. The TAR of the whole laser scan segment, ${\left\{\kappa_{i}\right\}}_{i=1}^{N}$, is normalized by dividing it by its absolute maximum value.

When the contour is traversed counterclockwise, positive, negative and zero values of TAR mean convex, concave and straight-line points, respectively.

**Figure 9.**
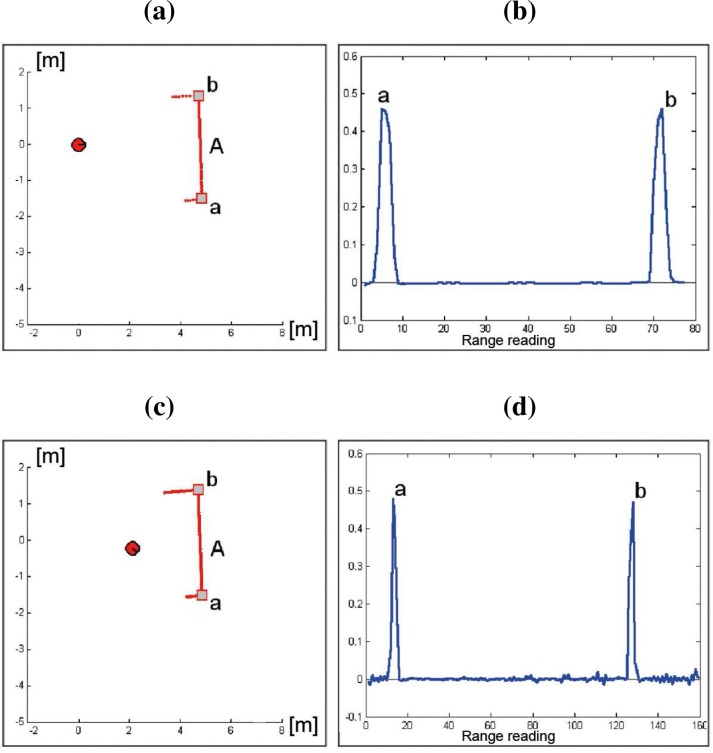
(a) Laser scan #1. (b) Adaptive TAR associated to scan segment A in (a). (c) Laser scan #2. (d) Adaptive TAR associated to scan segment A in (c).

Figure 9 shows two laser scan segments taken from different points of view. Figures 9b and 9d present the two adaptive TAR associated to Figures 9a and 9c, respectively. Although the number of range readings in the acquired segments are significantly different, both representations detect the same sets of dominant points.

**Figure 10.**
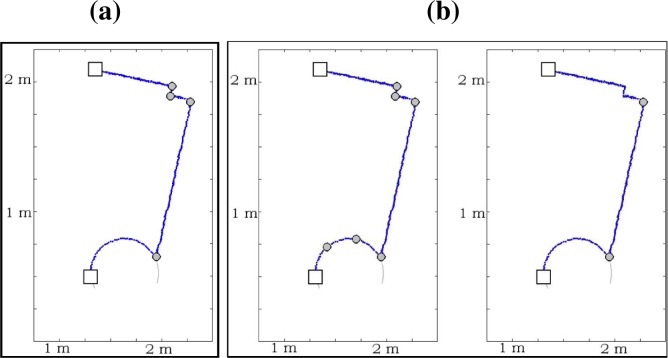
(a) Dominant points detected from the TAR obtained using an adaptive triangle side length; and (b) dominant points detected from the TAR obtained using a *t* value equal to 3 (left image) or *t* value equal to 15 (right image).

The advantage of measuring the curvature in an adaptive way can be appreciated in Figure 10. Figure 10a shows the dominant points detected from the adaptive TAR associated to the laser scan segment (triangle side lengths ranging from 3 to 15). The scheme to detect line and curve segments described in [16] has been used. It can be noted that all dominant points are correctly detected. On the contrary, Figure 10a,b shows the dominant points obtained by the same process when two constant triangle side length values are used. It can be appreciated that when a low value is used (*t* = 3), the TAR is too noisy and false dominant point detection occurs. On the contrary, if a high value is used (*t* = 15), the representation is excessively filtered, and some dominant points are lost.

### 4.3. Laser Scan Descriptor Under General Affine Transformations

Let ${\left\{x_{i},y_{i}\right\}}_{i=1}^{N}$ be the Cartesian coordinates of the set of range readings associated to a laser scan segment. If this scan segment is subjected to an affine transformation, the relation between the original and the distorted representations is given by

(15) $$\left[\begin{array}{c} {\hat{x}}_{i}\\ {\hat{y}}_{i} \end{array}\right]=\left[\begin{array}{cc} a & b\\ c & d \end{array}\right]\;\left[\begin{array}{c} x_{i}\\ y_{i} \end{array}\right]+\left[\begin{array}{c} t_{1}\\ t_{2} \end{array}\right]$$

where ${\left\{{\hat{x}}_{i},{\hat{y}}_{i}\right\}}_{i=1}^{N}$ is the affine distorted representation of the scan segment, *a*, *b*, *c* and *d* represent scale, rotation and shear and *t*_1_ and *t*_2_ represent translation. By substituting the expressions for ${\left\{{\hat{x}}_{i},{\hat{y}}_{i}\right\}}_{i=1}^{N}$ into Equation 13, we obtain

(16) $$\begin{array}{l} f_{{\hat{x}}_{i}}=a(x_{i+t_{f}\;[i]}-x_{i})+b(y_{i+t_{f}\;[i]}-y_{i})=a\cdot f_{x_{i}}+b\cdot f_{y_{i}}\\ f_{{\hat{y}}_{i}}=c(x_{i+t_{f}\;[i]}-x_{i})+d(y_{i+t_{f}\;[i]}-y_{i})=c\cdot f_{x_{i}}+d\cdot f_{y_{i}}\\ b_{{\hat{x}}_{i}}=a(x_{i-t_{b}[i]}-x_{i})+b(y_{i-t_{b}[i]}-y_{i})=a\cdot b_{x_{i}}+b\cdot b_{y_{i}}\\ b_{{\hat{y}}_{i}}=c(x_{i-t_{b}[i]}-x_{i})+d(y_{i-t_{b}[i]}-y_{i})=c\cdot b_{x_{i}}+d\cdot b_{y_{i}} \end{array}$$

Then, if we substitute 16 into 14, it is obtained that *κ̂_i_* = (*ad* − *bc*)*κ_i_*, where *κ̂_i_* is the affine transformed version of *κ_i_*. As the TAR is normalized by its maximum value, this representation is invariant to the affine transformations.

### 4.4. Experimental Results

**Figure 11.**
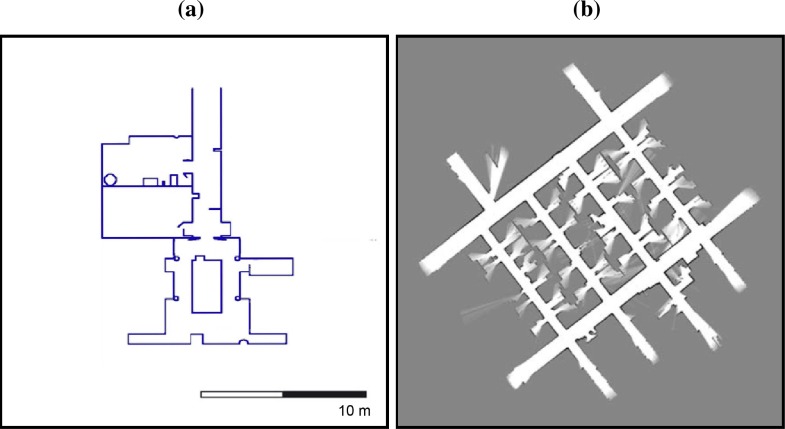
(a) The first test area, an office-like environment sited at the Technology Park of Andalusia (Málaga); and (b) the map of a part of the Intel Jones Farms Campus in Hillsboro, Oregon (source: the Radish repository http://radish.sourceforge.net/).

To evaluate the performance of the proposed algorithm, two laser scan datasets taken from two different environments have been selected: the fourth level of the University Institute of Research at the Technology Park of Andalusia in Málaga, Spain, and a part of the Intel Jones Farms Campus, Oregon. The first dataset has been collected using Rex, a Pioneer 2AT robot from ActivMedia equipped with a SICK LMS200. The field of view is 180° in front of the robot and up to 8 m distance. The range samples are spaced every half a degree, all within the same plane. Figure 11a shows the first test area. This test area is an office-like environment which presents a higher density of detected landmarks. In the second test area, the scan data are taken in the Intel Jones Farms Campus in Oregon. The ground map is shown in Figure 11b. The environment is also an office-like structure. This second dataset has been obtained from the Radish repository [40] and the robot platform for this dataset was a Pioneer2DX (odometry) with a SICK LMS200. It must be noted that the set of threshold values used by the algorithm are the same for both scenarios.

**Figure 12.**
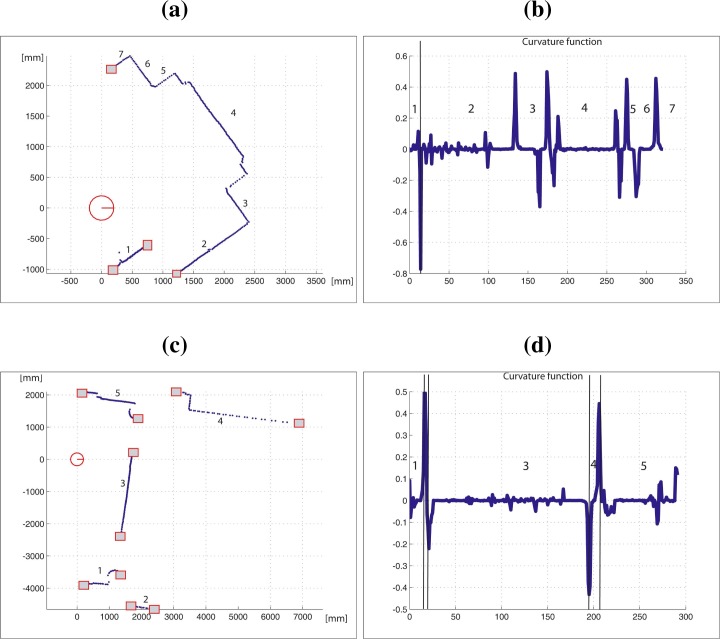
(a) Laser scan #3. (b) Curvature functions associated to (a). (c) Laser scan #4. (d) Curvature functions associated to (c).

**Figure 13.**
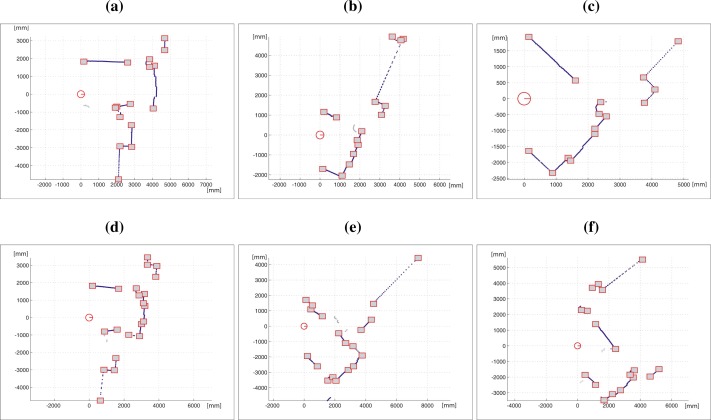
(a)–(f) Laser scan segmentations.

Figure 12 shows the detected segments at two different robot poses at the first scenario. Figures 12a and 12c present two scan data collected in these poses. The laser scan range readings has been marked as dots. The squares represent the start and end-points of each laser scan segment. Figures 12b and 12d show the curvature functions associated with the laser scans in Figures 12a and 12c, respectively. The segmented portions of the curvature functions are bounded by breakpoints or rupture points. Figure 13 shows the segmentation of several laser scans acquired by the robot in the second test area. It can be noted that all segments are correctly obtained.

Finally, in this work, due to the usual velocities in mobile robotics, the assumption of low speeds (i.e., few meters per second) has been taken. Therefore, the effect of the robot motion on individual range readings is negligible. Besides, the measured distance *r_i_* is perturbed by a systematic error and a statistical error, usually assumed to follow a Gaussian distribution with zero mean. In order to compensate the systematic error, it has been approximated by a sixth-order polynomial which fits the differences between the measured distance and the true obstacle distance in the least-squares sense (see [14] for further details).

## 5. Comparative Study

In order to compare the proposed method to other approaches, we have implemented several laser scan data segmentation algorithms. Particularly, for the purpose of comparison, the split-and-merge (SM) algorithm [20], the iterative-end-point-fit (IEPF) method [18], the split-and-merge fuzzy (SMF) [19], the curvature scale space (CSS) [32], a Hough-based algorithm [23] and an adaptive curvature approach (CUBA) [16] has been selected. The test database consists of 50 laser scans obtained from a set of 10 artificial maps that have been created using the Mapper3 software from Activmedia Robotics. Laser scans have been obtained from these maps using MobileSim. The aim of using these artificial maps is to test each algorithm in a controlled and supervised environment, where the number and shape of segments are known (ground truth). Simulated laser sensor exhibits statistical errors of *σ_r_* = 5 mm and *σ_φ_* = 0.1 degrees. Each test scan consists of 360 range readings and it represents several line and curve segments.

Algorithms are programmed in C, and the benchmarks are performed on a PC with a Pentium II 450 MHz. The minimum number of points per line or curve segment have been fixed to 10 and the minimum physical length of a segment have been fixed to 50 cm. Both parameters have been chosen according to the simulated scans. Other parameters are algorithm-specific.

Segmentation experiments were repeated several times to choose good values for each approach. Segment pairs are initially matched using a *χ*^2^-test with a matching valid gate value of 2.77 (75% confidence interval). Then, extracted segments are matched to true segments using a nearest-neighbor algorithm. Experimental results are shown in Table 1. The correctness in these methods can be measured as [13]

(17) $$\mathrm{TruePos}=\frac{\mathrm{NumberMatches}}{\mathrm{NumberTrueSeg}}$$

(18) $$\mathrm{FalsePos}=\frac{\mathrm{NumberSegExAl}-\mathrm{NumberMatches}}{\mathrm{NumberSegExAl}}$$

where *NumberSegExAl* is the number of segments extracted by an algorithm, *NumberMatches* is the number of matches to true segments and *NumberTrueSeg* is the number of true segments. To determine the precision, line and curve segments are taken into account. Line segments are characterized by *α*, the angle between the X axis and the normal of the line, and *d*, the perpendicular distance of the line to the origin. Then, the following two sets of errors on line parameters are defined:

(19) $$\begin{array}{l} \Delta d:\Delta d_{i}=\left|d_{i}-d_{i}^{t}\right|,i=1\ldots n\\ \Delta \alpha :\Delta \alpha_{i}=\left|\alpha_{i}-\alpha_{i}^{t}\right|,i=1\ldots n \end{array}$$

where *n* is the number of matched pairs, $d_{i}^{t}$ and $\alpha_{i}^{t}$ are line parameters of a true line, and *d_i_* and *α_i_* are line parameters of the corresponding matched line. It is assumed that error distributions are Gaussian. Then, the variance of each distribution is computed as

(20) $$\sigma_{\Delta d}^{2}=\frac{1}{n-1}\sum {\left({\Delta d}_{i}-\frac{1}{n}\sum {\Delta d}_{i}\right)}^{2}$$

Similar sets of errors on curve parameters are defined (*x_c_* and *y_c_* define the center of the circle and *ρ* is the radius). From Table 1, it can be noted that the IEPF and SM algorithms perform faster than the others. The proposed method is faster than the CUBA, CSS and HT approaches. Besides, the CSS, CUBA and the proposed algorithm are the only methods that do not split curve segments into short straight-line segments. Therefore, they have the best scores in term of correctness and precision with respect to curve segments.

**Figure 14.**
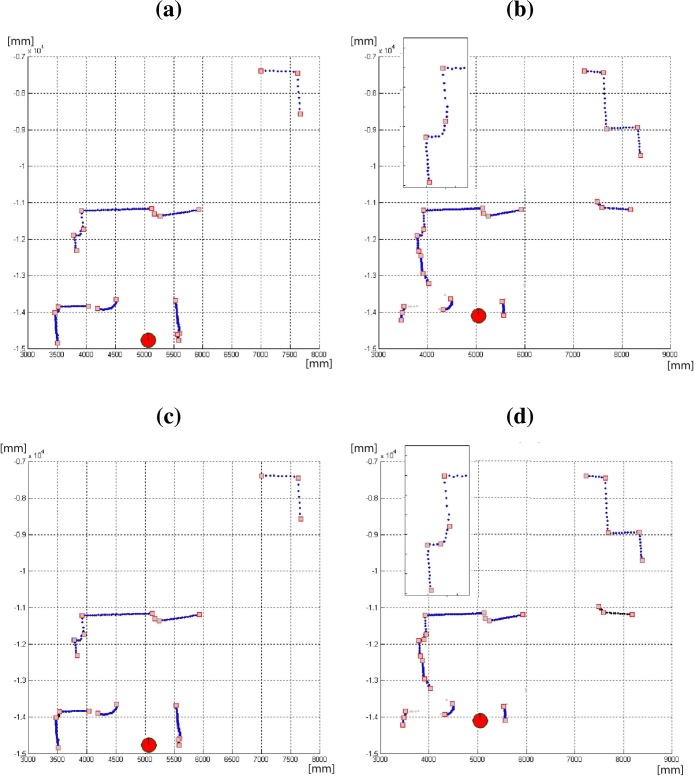
(a)–(b) Results of the proposed algorithm from different poses in the same indoor environment. The same results are provided by the algorithm from these robot poses (see details in the figure (b); (c)–(d) CUBA algorithm results from the same tests. The algorithm split the curve segment in different parts due to this algorithm is not invariant to robot pose.

Finally, a typical situation to illustrate the improvement in the proposed algorithm is shown in Figure 14. In this case it is compared to the CUBA algorithm (Figures 14c and 14d) in the same indoor environment but from different robot poses. The inset of Figure 14d shows a part of the scan in detail, where the algorithm split the curve segment in two parts. This is not the result obtained from the previous pose (Figure 14c), in this case the same part of the scan has been considered as only one curve segment. It can be noted how the proposed algorithm provides the same results from different poses (Figures 14a and 14b) due to its invariant properties. These situations arise when some parts of the environment are occluded or are out of the vision field of the laser scanner during some time, and are observed again from a different robot pose (translation and/or rotation).

## 6. Conclusions and Future Works

This paper presents a curvature-based environment description for robot navigation using laser range sensors. The main advantage of using curvature information is that the algorithm can directly provide line and curve segments, and improve the robustness against noise. Besides, this approach exhibits superior performance over traditional segmentation algorithms based on polygonal approximations which assume that the laser scan is only composed of line segments. The proposed segmentation module uses an adaptive estimate of the curvature according to a triangle-area representation, where the triangle side length at each range reading are adapted to the local changes of the scan data. The segmentation results are used to provide natural landmarks of the robot environment: line and curve segments, corners and edges. Finally, the segmentation algorithm has been compared with the state-of-the-art algorithms in terms of performance, robustness and speed. Although the proposed algorithm provides slightly better results than previously proposed curvature-based approaches (the best results has been obtained for curve segments, see Table 1), it is faster, invariant to translation and rotation, and robust against noise. Future work will focus on the development of an algorithm for scan matching of two consecutive scan data acquired by the sensor based on the extracted features and application in dynamic environments (e.g., people or object moving around the robot). This algorithm must be capable to estimate the robot pose by trying to maximize the overlapping between two sets of features extracted by the environment description proposed in this work.

## 7. Glossary

1. *Odometry:* is the use of data from the movement of proprioceptive sensor (actuators) to estimate change in the robot pose over time. Odometry is used by the current robots to estimate (not determine) their pose relative to an initial location.
2. *Localization:* is defined as the knowledge of the position and orientation of the robot in the working environment at every instant of time. In a local point of view, given a map of the environment and an initial pose (x - y position, and *θ* orientation), the localization task consists of tracking the mobile agent around the environment.
3. *Mapping:* is defined as the problem of acquiring a spatial model of a robot environment. Usually, mapping algorithms obtain an instantaneous local representation of the scene according to the current sensor reading, including static and dynamic objects. Next, a global map is built only with static objects.
4. *SLAM:* is a technique used by autonomous mobile robots to build up a map within an unknown environment while keeping track of their current position at the same time.
5. *Natural landmarks:* Landmarks are defined as features which are determined by the system and detected according to some criteria. In this situation, natural landmarks are directly selected in the natural scene considering their geometrical or photo-metrical features.
6. *Segmentation:* is a process of aiming to classify each scan data into several groups, each of which possibly associates with different structures of the environment.
7. *Breakpoints:* are scan discontinuities due to a change of the surface being scanned by the laser sensor.

## Figures and Tables

**Table 1.** Experimental results of several segmentation algorithms (see text).

Algorithm	SM	SMF	IEPF	CSS	HT	CUBA	Proposed
Execution time (ms)	7.1	14.6	4.2	39.1	33.4	13.6	10.1
TruePos	0.77	0.79	0.78	0.93	0.73	0.92	0.93
FalsePos	0.23	0.23	0.26	0.03	0.28	0.02	0.02
*σ_Δd_* [mm]	15.2	12.3	17.2	10.4	9.8	10.2	10.2
*σ*_Δα_ [deg]	0.82	0.63	0.79	0.58	0.58	0.60	0.59
*σ*_Δ*x*_*c*__ [mm]	14.1	13.1	13.9	10.1	13.0	9.8	9.7
*σ*_Δ*y_c_*_ [mm]	12.6	12.9	13.1	9.8	12.7	9.9	9.6
*σ*_Δ*ρ*_ [mm]	8.3	7.9	8.5	7.9	8.5	6.5	6.1
